# Inflammation Induced Sensory Nerve Growth and Pain Hypersensitivity Requires the N-Type Calcium Channel Cav2.2

**DOI:** 10.3389/fnins.2019.01009

**Published:** 2019-09-19

**Authors:** Saumitra Pitake, Leah J. Middleton, Ishmail Abdus-Saboor, Santosh K. Mishra

**Affiliations:** ^1^Department of Molecular Biomedical Sciences, College of Veterinary Medicine, North Carolina State University, Raleigh, NC, United States; ^2^Department of Biology, University of Pennsylvania, Philadelphia, PA, United States; ^3^Comparative Medicine Institute, College of Veterinary Medicine, North Carolina State University, Raleigh, NC, United States; ^4^The W.M. Keck Center for Behavioral Biology, North Carolina State University, Raleigh, NC, United States; ^5^Program in Genetics, North Carolina State University, Raleigh, NC, United States

**Keywords:** Cav2.2, inflammation, pain, nerve growth, hyperalgesia

## Abstract

Voltage-gated calcium channels (VGCCs) are important mediators of pain hypersensitivity during inflammatory states, but their role in sensory nerve growth remains underexplored. Here, we assess the role of the N-type calcium channel Cav2.2 in the complete Freund’s adjuvant (CFA) model of inflammatory pain. We demonstrate with *in situ* hybridization and immunoblotting, an increase in Cav2.2 expression after hind paw CFA injection in sensory neurons that respond to thermal stimuli, but not in two different mechanosensitive neuronal populations. Further, Cav2.2 upregulation post-CFA correlates with thermal but not mechanical hyperalgesia in behaving mice, and this hypersensitivity is blocked with a specific Cav2.2 inhibitor. Voltage clamp recordings reveal a significant increase in Cav2.2 currents post-CFA, while current clamp analyses demonstrate a significant increase in action potential frequency. Moreover, CFA-induced sensory nerve growth, which involves the extracellular signal-related kinase (ERK1/2) signaling pathway and likely contributes to inflammation-induced hyperalgesia, was blocked with the Cav2.2 inhibitor. Together, this work uncovers a role for Cav2.2 during inflammation, demonstrating that VGCC activity can promote thermal hyperalgesia through both changes in firing rates of sensory neurons as well as promotion of new neurite outgrowth.

## Introduction

Inflammation-induced pain hypersensitivity is a manifestation of increased sensory input, altered neurotransmitter release in the spinal cord, heightened excitability of neurons, and phenotypic changes brought about by afferent neurons innervating the site of inflammation (Woolf et al., 1997; Lankford et al., 1998). Release of cytokines and other inflammatory agents including bradykinin, prostaglandin E2, ATP, and a host of other agents at the site of inflammation collectively contributes toward lowering the excitability threshold of primary afferents near the affected area in the periphery (Gold and Flake, 2005; Miller et al., 2009; Fehrenbacher et al., 2012). A myriad of ionic mechanisms contribute to the increased sensory transduction observed during inflammation; these implicate changes in gene expression and voltage dependence of voltage-gated Na^+^, K^+^, and Ca^2+^ channels and ligand-gated ion channels such as transient receptor potential (TRP channels) and purinergic receptors (Xu and Huang, 2002; Amaya et al., 2006; Yu et al., 2008; Lu et al., 2010).

Several studies have demonstrated that voltage-gated calcium channels (VGCCs) play an important role in inflammation-induced hyperexcitability of primary afferents (Schaible et al., 2000; Zhang and Dougherty, 2014; Berta et al., 2017). Although almost all subtypes of VGCCs (N, P/Q, R, and T) are expressed throughout the central nervous system (Hillman et al., 1991; Westenbroek et al., 1992; Zamponi et al., 2015). Recent studies have highlighted the role that N-type (Cav2.2) channels play in neuropathic and inflammatory pain (Snutch, 2005; Altier et al., 2007; Lee, 2013). Cav2.2 channel is expressed abundantly in dorsal root ganglion (DRG) neurons and genetic and pharmacological strategies that block Cav2.2 activity are anti-nociceptive (Woolf et al., 1997; Lankford et al., 1998; Gold and Flake, 2005; Miller et al., 2009; Fehrenbacher et al., 2012). Importantly, intracellular calcium signaling has been shown to regulate neurite growth patterns in neurons through VGCC channels (Vigers and Pfenninger, 1991; Weiss, 2008). In PC12 neural cell line, calcium influx through VGCC causes extensive neurite outgrowth (Solem et al., 1995). In another study in parasympathetic cultured neurons, it has been shown that blockade of calcium influx through L-type and N-type VGCCs as well as transient receptor potential canonical (TRPC) channels, reduces the growth of neurite processes while release from intracellular stores was not significantly affected (Zamburlin et al., 2013). Additionally, *Xenopus* live imaging findings, coupled with studies of cultured *Xenopus* spinal neurons, demonstrate that an optimal frequency of calcium transients regulates neurite extension (Gu and Spitzer, 1995). However, it remains unclear whether this same phenomenon drives the afferent sensory growth in inflammation, which leads to pain hypersensitivity at the periphery, and furthermore which downstream signaling cascades are involved in sensory afferent outgrowth.

In this study, we investigated the role of Cav2.2 in sensory nerve outgrowth during an inflammatory pain state. By employing *in vitro* and *in vivo* cellular, pharmacological, behavioral, and physiological methods, we revealed a function for the Cav2.2 channel as a modulator of sensory nerve growth during inflammation-induced hyperalgesia. Selective blockade of Cav2.2 channels leads to a decrease in neurite outgrowth and an attenuation of thermal, but not mechanical pain. We demonstrate with RNAscope technology that thermal hyperalgesia during inflammation is likely mediated by Cav2.2 upregulation in TrpM8+,TrpV1+, and TrpA1+ sensory neurons. Based on these findings we provide a mechanism for CFA-induced inflammatory pain hypersensitivity; wherein Cav2.2 is the primary effector and ERK1/2-MAPK is the downstream target.

## Materials and Methods

### Animals

Six to eight-weeks old C57BL/6 mice of either sex were used for all the experiments and mice were purchased from the Jackson Laboratory. All procedures were conducted according to animal protocols approved by the university Institutional Animal Care and Use Committee (IACUC) at North Carolina State University and the University of Pennsylvania in accordance with National Institutes of Health (NIH) guidelines. All mice were group housed with four/five animals per cage.

### Drugs

ω-conotoxin GVIA (C9915) was purchased from Sigma-Aldrich and dissolved in phosphate buffered saline (PBS; pH 7.4). Nifedipine (481981) was purchased from Calbiochem and was dissolved in DMSO.

### Behavioral Assays

#### Thermal

A Hargreaves’ test apparatus (Ugo Basile) was used to measure thermal responses to radiant heat. Mice were placed in individual chambers for 10 min for habituation. A radiant heat source was focused on the paws and withdrawal latency was recorded (Mishra et al., 2011). Dry ice was used to assess the acute temperature sensitivity (Brenner et al., 2012). Each animal was tested at least twice to address the variability in responses.

#### Mechanical

Mechanical test was performed using a von-Frey apparatus (Ugo Basile). An electronic von-Frey filament was used to measure force in grams applied to the paw and the cutoff force was set at 50 g. Mice were habituated in the plexiglass chambers for 10 min prior to the experiment. Minimum of four measurements were taken from each paw, and the average force at which paw withdrawal was observed and reported accordingly (Mishra et al., 2011).

#### CFA-Induced Inflammation

Sterile complete Freund’s adjuvant (CFA; 20 μl) was injected into the plantar surface of the hind paw to induce inflammation. The site of injection and the surrounding area was observed 24 h post injection to visually confirm inflammation. Mice were then tested for thermal, cold, and mechanical pain sensitivity. As a control, PBS (20 μl) was injected into the plantar surface, and mice were then subjected to the same behavioral testing. The observer was blinded during all behavior recordings.

### Intrathecal Injection

Mice were anesthetized briefly under isoflurane. Cav2.2 inhibitor conotoxin (300 pmol/10 μl) and vehicle PBS (10 μl) were injected in between the L5 and L6 lumbar space using a 30-gauge insulin syringe, and a tail flick indicated successful entry of the needle into the subdural space. The injection was repeated every 24 h for 3 days. Baseline behavior was recorded immediately following intrathecal injection of vehicle and/or conotoxin and subsequent analyses were performed 24 and 72 h post-injection to assess the results of the specific treatments. All the behavior analyses were performed by an observer who was blinded to the treatment groups.

### Western Blot

To extract total protein, dorsal root ganglia, paws, and sciatic nerves were homogenized using a tissue homogenizer in the presence of 100 μl of ice cold RIPA buffer supplemented with protease inhibitor tablets ı(Pierce^TM^). Total protein of lysates was measured using standard BCA (Bicinchoninic Acid Assay). Protein lysates were then denatured by heating at 95°C in Laemmli’s buffer containing 2% w/v SDS, 62.5 mM Tris (pH 6.8), 10% glycerol, 50°mM DTT, and 0.01% w/v bromophenol blue. The lysates were cooled on ice and briefly micro-centrifuged. Aliquots of 35°μg of protein were loaded onto a 4–12% SDS-PAGE gel, and subsequently electro blotted onto PVDF membranes. Membranes were incubated in 15 ml of blocking buffer [20 mM Tris base and 140 mM NaCl, 5% bovine serum albumin (BSA), and 0.1% Tween-20] for 1 h. Membranes were then incubated with the desired primary antibody diluted in 10 ml of blocking buffer at 4°C overnight. Next day membrane was washed and incubated with an appropriate horseradish peroxidase-conjugated secondary antibody (1:1000) to detect proteins in 10°ml blocking buffer for 1 h at room temperature. Immuno-reactive proteins were revealed using enhanced chemiluminescence detection (Pierce ECL^TM^). Anti-TUJ1 (Cat. MMS-435P) was purchased from Covance, Anti-Cav2.2 (Cat. sc-271010), Anti-GAPDH (Cat. sc-32233) were purchased from Santa Cruz Biotechnology. Anti-ERK 1/2 (Cat. 4695) and Anti-phospho-ERK 1/2 (Cat. 4370) were purchased from Cell Signaling technology. All primary antibodies were used at a dilution of 1:100 except for Anti-Cav2.2 which was used at a dilution of 1:100. Secondary anti-rabbit and anti-mouse antibodies were purchased from Santa Cruz Biotechnology. Alexa 488 and Alexa 555 conjugated secondary antibodies were purchased from Thermo Fisher Scientific. The intensity of each protein band was analyzed using an opensource software ImageJ (NIH). The expression of protein was normalized to the expression of loading control GAPDH. For phosphorylated ERK 1/2 expression, Total extracellular signal-related kinase (ERK) was used as a control.

### Immunohistochemistry/Immunocytochemistry

Sciatic nerve were dissected from mice and quickly frozen in Tissue-Tek^®^ O.C.T. compound over dry ice. 10–12°μM sections were cut using cryostat and were placed on charged slides. The sections were then fixed using 4% Paraformaldehyde solution for 20 min and then quickly rehydrated with PBS for 5 min. Sections were then incubated in a 5% BSA solution containing 0.1% Triton for better antigen exposure for 45 min. The sections were then washed with PBS and incubated with 5% BSA solution with desired primary antibodies at 4°C overnight. Alexa conjugated secondary antibodies were used along with DAPI stain containing mounting medium and sections were imaged using Leica DM5000b microscope. For immunocytochemistry, mice were unilaterally injected in the hind paw with CFA, and ipsilateral and contralateral lumbar DRGs were harvested 24 h later. To assess the effect of conotoxin on neurite growth and Cav2.2 expression, ipsilateral DRGs from control (vehicle treated), CFA and CFA + conotoxin treated mice were harvested after 24 h. DRGs were then dissociated into single cells and plated at equal density on glass coverslips. The cells were fixed with 4% paraformaldehyde for 15 min and then quickly rehydrated with PBS for 5 min. Immunostaining was performed as described above using antibodies against TUJ1 (1:1000) and Cav2.2 (1:1000).

### RNAScope *in situ* Hybridization

C57BL/6 mice were injected with CFA (20 μl) unilaterally in the right hind paw and ipsilateral and contralateral lumbar dorsal root ganglia (L4-L5) were harvested 48 h post treatment. Dorsal root ganglia were quickly frozen over dry ice in Tissue-Tek^®^ O.C.T. 10–12 μM sections were cut using a cryostat (Leica CM3050S) and were placed on charged slides. The sections were further processed according to RNAscope^®^ Multiplex Fluorescent Assay v2 (Advanced Cell Diagnostics, Hayward, CA, United States) protocol using target probes for Cav2.2, TRPV1, TRPM8, TRPA1, MrgprD, and MrgprB4. RNAScope results were examined using Leica DM6000FS confocal microscope at 20× magnification. Fifteen cells with detectable signal were selected at random for quantification by a blinded observer who was unaware of the treatment groups. The signal intensity of mRNA clusters observed within each cell was analyzed by drawing a region of interest (ROI) around each cell and mean signal intensity in arbitrary units generated by ImageJ software was noted. The dimensions of the ROI were kept constant throughout the analysis to avoid bias. This process was repeated for each channel (red, green, and blue) including overlay images. At least 15 ROIs were drawn per section and at least five ipsilateral and five contralateral sections were analyzed from *n* = 3 mice. The entire quantification was performed by an observer in a manner blinded to mRNA probes and channel assignments.

### Electrophysiology

#### Spontaneous Action Potential Recording in Dissociated DRG Neurons

Whole cell current clamp recording was carried out in dissociated DRG neurons after plating them overnight. Thin walled borosilicate glass capillaries (TW150-4) were purchased from World Precision Instruments and electrodes were pulled using Sutter P-97 puller. The pipette resistance was between 2 and 3 MΩ in bath solution. For current-clamp recording, the internal solution contained 145 mM K-gluconate, 2 mM MgCl_2_, 1 mM CaCl_2_, 5 mM K_2_GTP, 5 mM HEPES, and 10 mM EGTA adjusted to pH 7.4 with KOH and the bath solution contained 140 mM NaCl, 5 mM KCl, 2 mM CaCl_2_, 1 mM MgCl_2_, 10 mM HEPES, and 10 mM Glucose adjusted to pH 7.4 with NaOH. Electrophysiologic recording was initiated in voltage clamp mode until attaining a stable whole-cell clamp and then switched to current clamp mode. DRG neurons were held at 0 pA, and the spontaneous action potentials were recorded in “zero current clamp” mode. Number of spontaneous action potentials observed in a fixed time interval were used to assess hypersensitivity between different treatment groups. Spontaneous action potentials were recorded from small diameter (<45 μM) DRGs dissociated from the lumbar region. The diameter was determined using 20× Nikon objective and scale generated by Nikon Elements software. Only neurons with a resting membrane potential of at least −40 mV and stable baselines were used for further experiments and analysis. Recordings were included only if the above-mentioned criteria were met. This was done to minimize spontaneous firing caused by deterioration or other artifacts during recording.

#### Cav2.2 Current Recordings

Internal solution for voltage clamp recordings contained (in mM): 140 Cs-Aspartate, 10 Cs2EGTA, 5 MgCl_2_, and 10 HEPES, pH 7.4. To isolate N-type Ca_2_+ currents, the K+ currents and L-type Ca^2+^ currents were blocked with tetraethylammonium-Cl (TEA-Cl) and 1 μM nifedipine (Calbiochem), respectively. The standard external solution was composed of (in mM): 137.5 TEA-Cl, 10 CaCl_2_, 10 HEPES, pH 7.4. Electronic compensation was used to reduce the effective series resistance (<5 MΩ). Currents were filtered at 2.9 KHz using a 4-pole Bessel filter. In most of the recordings, a 1 s prepulse to −20 mV followed by a 50 ms repolarization to −50 mV was administered before the test pulse to inactivate T-type calcium channels. I–V relationships were generated by applying 13 pulses from a holding potential of −70 mV in 10 mV increments. Pulse duration was fixed at 250 ms. Cav2.2 currents were recorded at their peak activation, at +10 mV, for all groups. Cell capacitance (pF) was used to normalize currents observed (pA) at +10 mV for all groups and the resulting current density (pA/pF) was used for statistical comparisons. All recordings were made at room temperature. All analyses were carried out using Clampfit software and only small-medium diameter (<45 μM) DRG neurons were used for recordings.

### Quantification and Statistical Analysis

Mice were randomly assigned to treatment groups. Behavior and *in situ* hybridization analyses were performed in a manner blinded to treatment assignments. Data are expressed as mean ± standard error of mean (SEM) and analyzed using GraphPad Prism 7.0. When comparing two treatment groups to one another student’s test was used. One-way ANOVA was applied to analyze repeated measurements from behavioral tests followed by Dunn’s *post hoc* test for multiple comparison. One-way ANOVA with Holm–Sidak test was applied to analyze electrophysiology and western blot data. All statistical tests were two-tailed, and the level of significance was set at *p* < 0.05.

## Results

### Co-expression of Cav2.2 and Nociceptive Markers in Normal and Inflammatory Conditions

**FIGURE 1:**
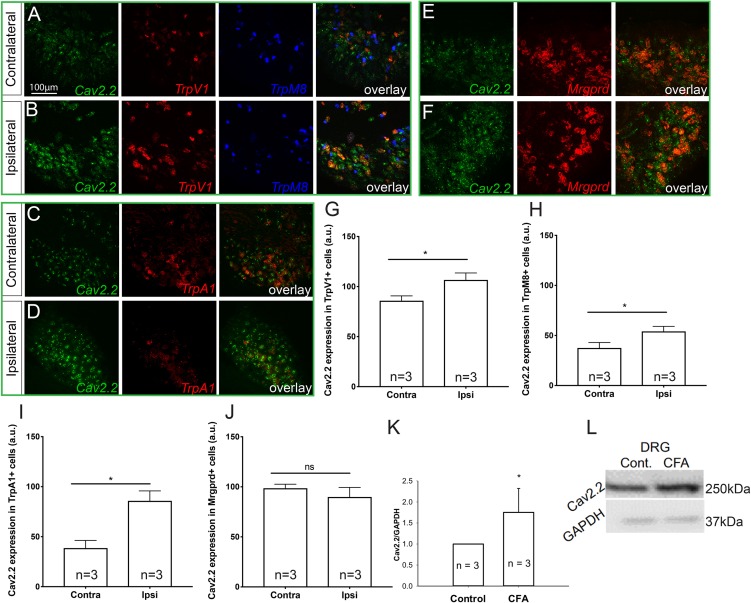
Cav2.2 RNA and protein are upregulated in thermal and not mechanical sensitive nociceptors after CFA-induced inflammation. *In situ* hybridization with gene specific RNAscope probes of contralateral (non-treated) **(A,C,E)** and ipsilateral (treated) **(B,D,F)** DRGs showing Cav2.2 mRNA (green) in heat-sensing TRPV1 + nociceptors (red), cold-sensing TRPM8 + nociceptors (blue), noxious-stimuli sensing TRPA1 + nociceptors (red), and mechanical-sensing MrgprD + nociceptors (red) observed 48 h post CFA induced inflammation. **(G–J)** Mean relative fluorescence intensity of Cav2.2 mRNA (expressed in “arbitrary units” defined by NIH/ImageJ software) in TRPV1+, TRPA1+, TRPM8+, and MrgprD + nociceptors in ipsilateral and contralateral DRGs. A significant increase in Cav2.2 mRNA in neuronal populations co-expressing TRPV1, TRPA1, and TRPM8, but not MrgprD mRNA, was observed after inflammation. *N* ≥ 5 sections per group obtained from three mice. ^∗^*P* < 0.05 comparing ipsilateral to contralateral DRGs with student’s *t*-test. Error bars represent standard error of the mean. **(K,L)** An increase in total Cav2.2 protein expression was observed in ipsilateral lumbar DRGs after CFA induced inflammation. ^∗^*P* < 0.05 comparing ipsilateral to contralateral DRGs with student’s *t*-test. Error bars represent standard error of the mean.

To determine which molecular populations of nociceptors might alter their expression of Cav2.2 during CFA-induced inflammatory states, we used RNAscope technology to perform *in situ* hybridization for *Cav2.2* and a panel of nociceptive markers. To induce inflammation, we performed unilateral intra-plantar injections of CFA, and 48 h post-injection we performed both two- and three-color *in situ* hybridization with RNAscope probes for *Cav2.2* in conjunction with *TrpV1*, *TrpM8*, and *TrpA1*, which are markers of thermal (hot and cold) and noxious stimuli, respectively (Figures 1A–D). Comparing the ipsilateral CFA-injected side to the control contralateral side, we observed a statistically significant increase in *Cav2.2* expression in *TrpV1*+, *TrpM8*+, and *TrpA1*+ nociceptors (Figures 1G–I). Conversely, such an increase was not observed when we performed two-color *in situ* hybridization with RNAscope probes for *Cav2.2* and *Mrgprd*, which is a marker for mechanosensitive non-peptidergic nociceptors (Figures 1E,F,I). Consistent with this finding, we did not observe significant co-labeling of *Cav2.2* with *Mrgprb4* C-fiber low-threshold mechanoreceptors in the ipsilateral or contralateral side post-CFA (Supplementary Figure S1). Taken together, these results are consistent with a role for Cav2.2 in thermal, but not mechanical hyperalgesia. The RNAscope results are consistent with increases in Cav2.2 protein expression during inflammation, and we further confirmed this by performing western blot analyses with an antibody specific to Cav2.2, and we observed a statistically significant increase in protein expression comparing the control (contralateral) versus CFA (ipsilateral) injected mice (Figures 1K,L).

### Conotoxin Inhibits CFA-Induced Thermal, but Not Mechanical Hyperalgesia

**FIGURE 2:**
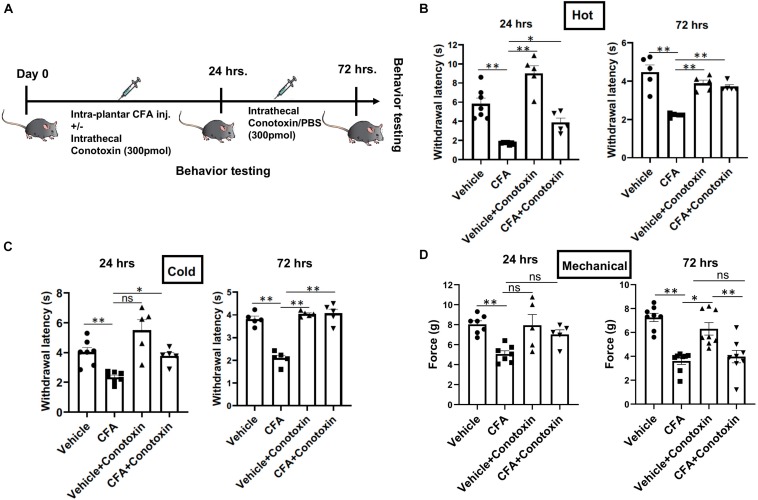
Effects of ω-conotoxin on inflammation induced pain behavior. **(A)** Shows a schematic representation of the behavior testing paradigm. Baseline thermal pain, acute cold pain, and mechanical pain sensitivities were recorded before CFA injection. CFA (20 μl) was injected intra-plantar in 6–10-week-old C57Bl6 mice with intrathecal ω-conotoxin (300 pmol/10 μl) and/or PBS (10 μl). Acute thermal pain (Hargreaves’), cold pain (Dry Ice), and mechanical pain (von-Frey) were recorded 24 and 72 h post CFA injection. **(B)** Inflammation induced thermal hyperalgesia was analyzed by Hargreaves’ test, **(C)** dry ice test, and **(D)** Mechanical hyperalgesia was assessed by von-Frey apparatus, *n* ≥ 5 mice, ^∗^*P* < 0.05, ^∗∗^*P* < 0.01. All statistical tests were performed as appropriate using a one-way ANOVA followed by Dunn’s *post hoc* test for multiple comparisons within a given behavioral test and time point (either 24 or 72 h).

Next, we sought to determine if blocking Cav2.2 action with ω-conotoxin, a potent inhibitor or Cav2.2, could prevent the development of hyperalgesia *in vivo*. Responsiveness to heat, cold, and mechanical stimuli were recorded during basal conditions and 24 and 72 h following unilateral CFA injection into the hind paw (Figure 2A). Consistent with prior studies, we observed hypersensitivity to heat, cold, and mechanical stimuli in CFA-injected mice compared to control vehicle injections (Figures 2B–D). To assess the inhibitory impact of conotoxin in attenuating this hypersensitivity, mice were administered either PBS or conotoxin intrathecally every 24 h for 3 days prior to behavioral assays (Figure 2A). We found that conotoxin administration reduced heat hyperalgesia in the Hargreaves’ thermal test at 24 and 72 h, preventing the reduced withdrawal latency observed with CFA alone (Figure 2B). Conotoxin also reduced cold hyperalgesia in the dry ice thermal test at 24 and 72 h, preventing the reduced withdrawal latency observed with CFA alone (Figure 2C). Next, we tested conotoxin’s effects on CFA-induced mechanical hypersensitivity using von Frey hair filaments and measuring the mechanical force threshold as a behavioral readout (Figure 2D). In this mechanosensitive paradigm, unlike our results with thermal hyperalgesia, we noticed that conotoxin treatment was unable to block nociceptive hypersensitivity to mechanical stimuli at either timepoint. Together, these behavior results implicate a role for the Cav2.2 channel in thermal, but not mechanical hyperalgesia coincident with inflammatory states.

### Electrophysiological Measurement of Sensory Neurons From Normal and Inflammatory Conditions

**FIGURE 3:**
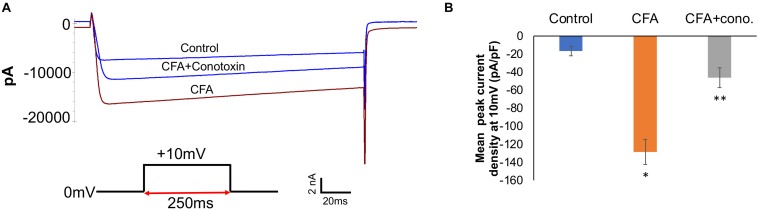
Complete Freund’s adjuvant induced inflammation leads to increased calcium currents via Cav2.2. **(A)** Overlay of representative raw Cav2.2 current traces from contralateral (control), ipsilateral (CFA-injected right side), and CFA + ω-conotoxin treated ipsilateral DRGs at +10 mV. **(B)** Quantification of peak current densities at +10 mV for all groups. A significant increase in calcium currents can be seen upon CFA treatment which was diminished post ω-conotoxin treatment, *N* ≥ 8 cells per group obtained from three mice per group, ^∗^*P* < 0.05 compare to control, ^∗∗^*P* < 0.01 compared to CFA, using one-way ANOVA followed by Holm–Sidak testing for multiple comparisons.

**FIGURE 4:**
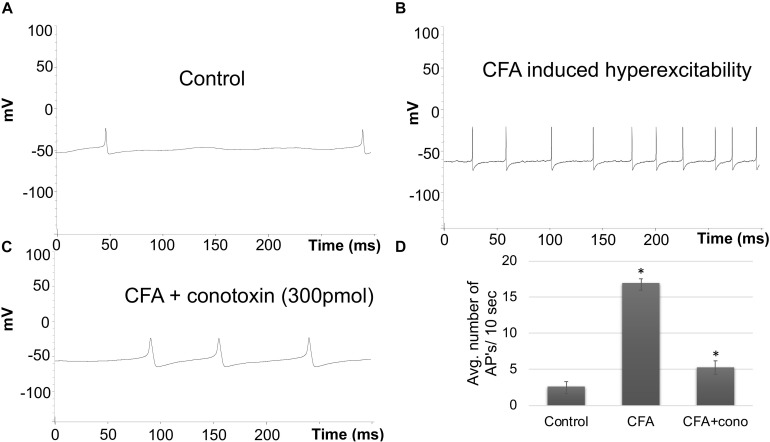
Effects of CFA and ω-conotoxin on spontaneous action potentials in DRGs. **(A)** Representative raw trace of spontaneous action potentials induced by changes in membrane potential in contralateral (non-treated) lumbar DRGs. **(B)** Representative raw trace showing an increase in spontaneous firing in ipsilateral DRGs post CFA-induced inflammation. **(C)** Representative raw trace showing a reduction in spontaneous firing behavior of DRGs post CFA-induced inflammation with the addition of conotoxin treatment. **(D)** Quantification of spikes per 10 s time interval, *N* ≥ 18 cells per group obtained from *n* = 3 mice per group, ^∗^*P* < 0.05, using one-way ANOVA followed by Holm–Sidak testing for multiple comparisons.

To complement the behavioral experiments and gain insight into the physiological properties of sensory neurons in relation to Cav2.2 during inflammatory conditions, we performed a panel of electrophysiological experiments in culture. Voltage clamp analyses revealed a significant increase in Cav2.2 current density in *ex vivo* conditions (Figures 3A,B). The specificity of these current recordings was established by using the selective blocker of Cav2.2, conotoxin (300 pmol), and this resulted in a significant reduction in current recordings (Figure 3). To assess the contribution of Cav2.2 channels to neuronal excitability, ipsilateral and contralateral lumber DRGs post CFA inflammation were isolated and spontaneous action potentials were recorded in current clamp mode (Figure 4A). Contralateral DRGs were unaffected by CFA-induced inflammation and rarely generated spontaneous action potentials while a significant increase in spontaneous action potentials was observed in ipsilateral DRGs (Figures 4B,D), and this was reduced significantly by conotoxin application (Figures 4C,D). These results demonstrate that CFA alters the intrinsic firing patterns of sensory neurons during inflammation in a Cav2.2 dependent manner.

### Increase in Sensory Nerve Density During Inflammation

**FIGURE 5:**
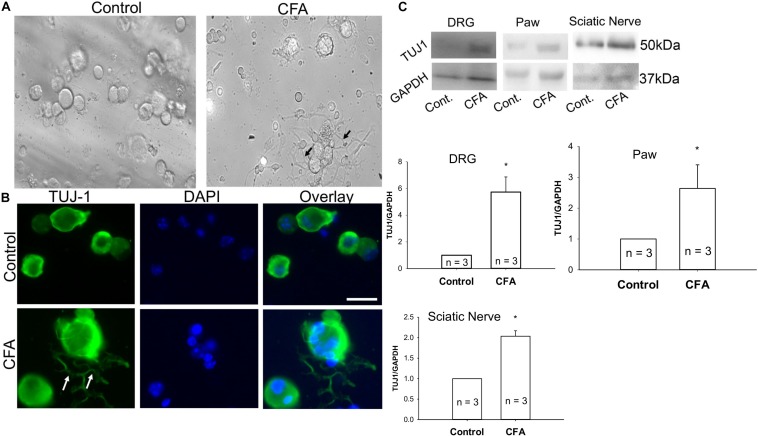
Complete Freund’s adjuvant induced inflammation leads to increased neurite growth. **(A)** Representative phase contrast image of dissociated dorsal root ganglia (DRG) in culture. Image on the left shows DRGs isolated from the contralateral side and image on the right shows DRGs isolated from the CFA-injected ipsilateral side 24 h following the injection. An increase in the neurite growth pattern post inflammation is indicated by black arrows. **(B)** Representative micrographs show an increase in neurite growth indicated by white arrows, as observed with TUJ1 immunostaining in ipsilateral versus contralateral DRGs. *N* ≥ 5 images per group obtained from three mice per group. Scale bar = 30 μM. **(C)** Immunoblots showing an increase in TUJ1 total protein expression in paws, DRGs and sciatic nerves, *n* = 3 mice per group, ^∗^*P* < 0.05 comparing control to CFA injected using student’s *t*-test.

To examine additional alterations of sensory neurons during inflammation that may require Cav2.2, mice were unilaterally injected in the hind paw with CFA, and ipsilateral and contralateral lumbar DRGs were harvested 24 h later. A phase contrast image of ipsilateral DRGs from CFA-injected mice revealed a significant increase in neurite formation (Figure 5A). We then used immunohistochemistry to identify sensory neurites by using the neuronal marker TUJ1, and we observed a significant increase in neuronal branching and growth only in DRG neurons ipsilateral to the CFA injection (Figure 5B). Next, we examined TUJ1 expression via western blot with protein extracted from the hind paw, DRG, and the sciatic nerve and found a significant increase in TUJ1 expression in all tissues examined (Figure 5C). Overall, our data reveals an increase in the sensory nerve outgrowth during inflammatory conditions.

### Inhibiting Cav2.2 Activity Blocks Sensory Neurite Outgrowth During Inflammation

**FIGURE 6:**
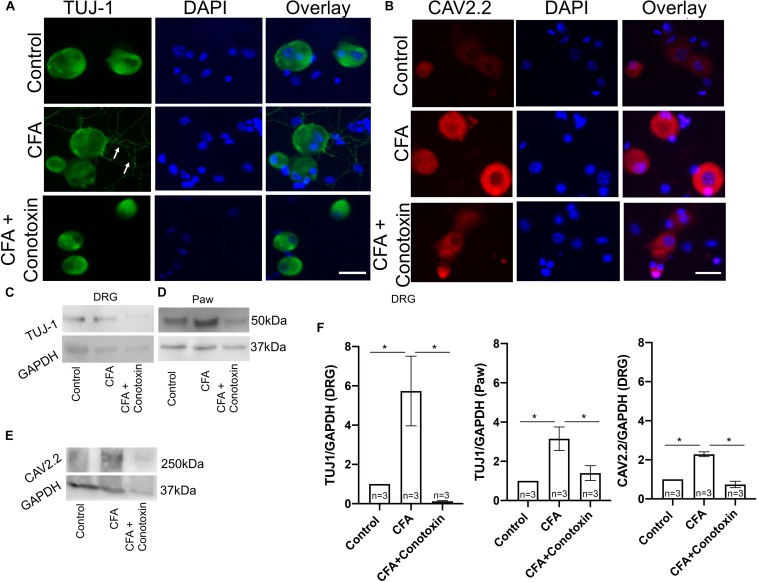
Effects of ω-conotoxin on peripheral neuronal growth and Cav2.2 protein expression. **(A)** Representative micrographs show TUJ1 immunolabeling in DRGs. A significant increase in TUJ1 labeled neurites can be seen post CFA inflammation (white arrows), and this is not observed post ω-conotoxin treatment. **(B)** Representative micrographs show an increase in Cav2.2 protein expression post CFA as revealed by antisera detected against Cav2.2 CFA. This increase in Cav2.2 expression is not observed when combining CFA with ω-conotoxin treatment. *N* ≥ 5 images per group obtained from three mice. Scale bar = 30 μM. **(C,D)** Immunoblots show an increase in TUJ1 protein expression in DRGs and paws. **(E)** Immunoblots show an increase in Cav2.2 protein expression post CFA treatment in DRGs, which is not observed combining CFA with ω-conotoxin treatment. **(F)** Quantification of TUJ1 and Cav2.2 immunoblots, *n* = 3 mice per group, ^∗^*P* < 0.05 using one-way ANOVA followed by Holm–Sidak testing for multiple comparisons.

To determine if Cav2.2 activity is necessary for the neurite outgrowth which we observed after CFA-induced inflammation, we harvested ipsilateral DRGs from mice treated with vehicle, CFA and CFA + conotoxin after 24 h and performed TUJ1 and Cav2.2 immunolabelling on dissociated cells (Figure 6A). Our immunostaining results using TUJ1 as a sensory neurite marker determined that Cav2.2 is required for the observed sensory neurite outgrowth phenotype (Figure 6A). Consistent with results described above in Figure 1, as well as published findings (Lu et al., 2010), we observed an increase in Cav2.2 protein expression in dissociated sensory neurons following CFA paw injection (Figure 6B). We further quantified the levels of Cav2.2 and TUJ1 proteins by western blotting in CFA and control mice, and we found an increase in TUJ1 expression, consistent with previous findings (Figure 5), upon CFA inflammation (Figures 6C–E). Interestingly, we observed a decrease in the level of TUJ1 (in the DRG and in the paw) and Cav2.2 (in the DRG) in mice 24 h after intrathecal administration of conotoxin (Figures 6C–F). Taken together, these data provide evidence for the requirement of Cav2.2 in CFA induced sensory neurite outgrowth.

### Extracellular Signal-Related Kinase Signaling Involved in Sensory Nerve Growth During Inflammation

**FIGURE 7:**
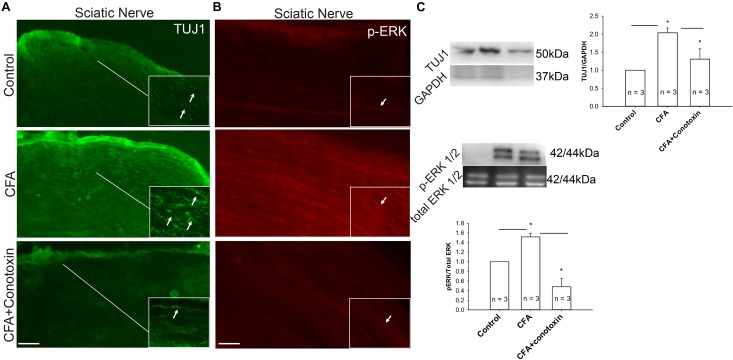
Inflammation associated neurite growth is dependent on Cav2.2 activity and is driven via ERK activation. **(A)** Representative micrographs show sciatic nerve sections from control, CFA treated and CFA + ω-conotoxin treated mice. A significant increase in TUJ1 expression can be seen post CFA treatment (white arrows in inset pointing at TUJ1 labeled afferents). ω-conotoxin prevents CFA mediated increase in TUJ1 labeled afferents. *n* = 3 mice per group. Scale bar = 30 μM, inset scale bar = 40 μM. **(B)** Representative micrographs show phosphorylated-ERK1/2 immunolabeling. Conotoxin prevents CFA induced increase in phosphorylated ERK. **(C)** Immunoblots showing changes in TUJ1and pERK1/2 expression in sciatic nerve consistent with histochemical analysis. Conotoxin mitigates TUJ1 and p-ERK1/2 activity upon CFA induced inflammation, *n* = 3 mice per group, ^∗^*P* < 0.01 using one-way ANOVA followed by Holm–Sidak testing for multiple comparisons between groups.

Several reports implicate the involvement of the ERK signaling pathway in neurite growth (Sgambato et al., 1998; Chen et al., 2011). We therefore sought to determine if this pathway might likewise be involved with Cav2.2-dependent sensory nerve growth during inflammation. Our immunohistochemical analysis revealed a CFA-induced upregulation of both the neuronal marker TUJ1 and the active phosphorylated form of ERK in the sciatic nerve (Figures 7A,B). Moreover, we observed that when conotoxin was injected intrathecally, post-CFA intra-plantar injection, it inhibited the CFA mediated increase in TUJ1 expression and ERK phosphorylation (Figures 7A,B). Further quantification by western blot was consistent with our immunohistochemistry results (Figure 7C). Overall, our data strongly suggest that Cav2.2 is required for CFA-induced sensory nerve growth through indirect phosphorylation of the signaling molecule ERK. Along these lines, ERK phosphorylation likely promotes the expression of genes which control the sensory neurite overgrowth phenotype.

## Discussion

**FIGURE 8:**
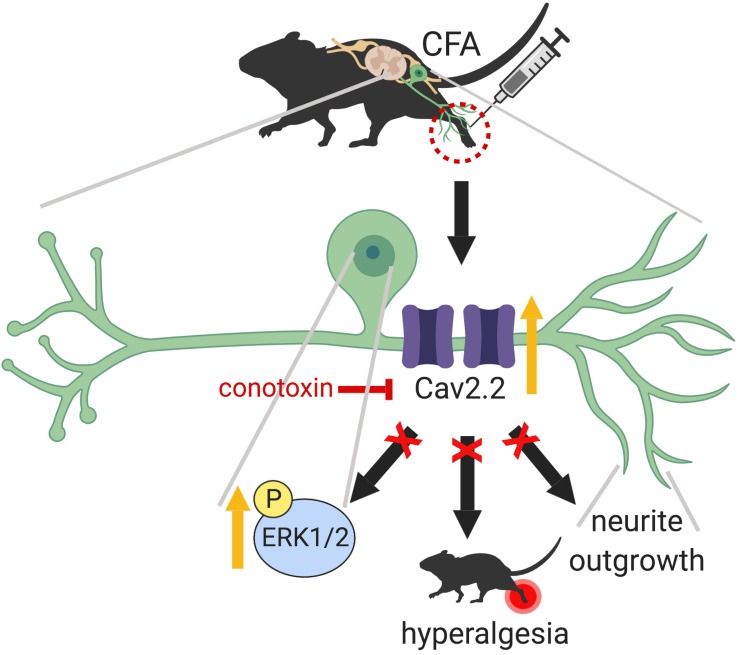
Graphical abstract summarizing the main findings of this work which highlight a peripheral role for Cav2.2 during inflammation. CFA induced inflammation leads to increased Cav2.2 expression and ion channel activity which seem to drive peripheral afferent growth at the site of inflammation. One way that these changes could be manifesting is through activation of the ERK1/2-MAPK pathway further downstream. Together, these changes contribute to inflammation associated hyperalgesia. Intrathecal injection of ω-conotoxin alleviates hyperalgesia by blocking Cav2.2 activity and associated afferent growth by downregulating ERK1/2. Schematic representation created with BioRender.com.

In the present study, we demonstrate that peripheral sensory nerve growth mediated via Cav2.2 activity drives inflammation-induced pain hypersensitivity. This conclusion is based on three lines of evidence. First, peripheral inflammation upregulates the expression of TUJ1, a post-mitotic neuritogenesis marker (von Bohlen, 2007) and Cav2.2 in the dorsal root ganglia. Second, pharmacological blockade of Cav2.2 channels attenuates inflammatory pain behavior in mice by preventing peripheral afferent growth. Third, inhibition of Cav2.2 results in downregulation of ERK1/2-MAP kinase, one of the major nociceptive signaling pathways associated with chronic inflammatory pain (Ji et al., 2002). Although persistent inflammation has been shown to alter density and distribution of a variety of voltage gated calcium channels (VGCCs) in the DRG (Lu et al., 2010), it remained unclear if VGCCs played any role in nerve growth post inflammation. Data from this study demonstrates that selective blockade of Cav2.2 with ω-conotoxin alleviates the inflammatory pain hypersensitivity by preventing afferent growth and ERK1/2 activation (Figure 8).

Recent studies have shown that injection of inflammatory compounds, such as carrageenan and CFA leads to upregulation of voltage gated sodium channels (Nav), TRP channels, acid-sensing ion channels (ASIC), and VGCCs in DRG neurons (Sgambato et al., 1998; Ji et al., 2002; von Bohlen, 2007; Yu et al., 2008). In particular, increased expression of TRPV1, TRPA1, and TRPM8 in primary afferents has been shown to be directly responsible for thermal and mechanical hypersensitivity in persistent inflammation (Mishra and Hoon, 2010; Chen et al., 2011; Mishra et al., 2011). N-type calcium channels have also been shown to play a major role in mediating release of synaptic transmitters like glutamate, acetylcholine, gamma-aminobutyric acid and calcitonin gene-related peptide (CGRP) (Nakanishi et al., 2010; Lalisse et al., 2018). Immunohistochemical evidence has also demonstrated increased localization of calcium channel subtypes in the dorsal horn of the spinal cord, nerve terminals and interneurons following injury and inflammation (Vigers and Pfenninger, 1991; Westenbroek et al., 1998; Cizkova et al., 2002). In addition, increased overlap of TRPV1+ and TRPA1+ sensory neurons in DRG with known pain neurotransmitters, such as substance P and CGRP post peripheral inflammation has been extensively reported and linked to thermal hyperalgesia (Nakanishi et al., 2010; Lalisse et al., 2018; Yu et al., 2008). Using RNA *in situ* hybridization, here we show a significant increase in Cav2.2 expression specifically in TRPV1+, TRPA1+, and TRPM8+ neuronal populations in ipsilateral DRGs 48 h post CFA-induced inflammation. Consistent with increased mRNA, total Cav2.2 protein expression was also upregulated in ipsilateral DRGs compared to contralateral DRGs. Together, these findings suggest that upregulation of Cav2.2 induced by peripheral inflammation might in turn cause increased TRP channel activity leading to further exacerbation of the condition. Interestingly, Cav2.2 was not upregulated in non-peptidergic mechanosensitive nociceptors labeled by MrgprD (Dong et al., 2001; Lembo et al., 2002; Cavanaugh et al., 2009), or MrgprB4+ C-low threshold mechanoreceptors post CFA-injection. These cell-type specific gene expression changes observed with Cav2.2 during inflammation suggest that other VGCCs besides the N-type may contribute to mechanical allodynia observed during inflammation.

In regards to loss-of-function studies with Cav2.2, it has been reported that the genetic ablation of Cav2.2 or α1B (the pore forming subunit of Cav2.2) in mice, results in attenuation of chronic inflammatory pain induced by formalin and acetic acid (Hatakeyama et al., 2001; Kim et al., 2001). Although the overall anti-nociceptive effects of Cav2.2 ablation on thermal hyperalgesia are consistent, there are conflicting reports about the reduction in mechanical pain hypersensitivity mediated via Cav2.2 (Hopkins et al., 1995; Rigo et al., 2013). This seeming inconsistency could be due to the diversity in the expression pattern of calcium channels or unintended consequences of the specific genetic strategies used for ablation (Olivera et al., 1994). As an alternative to genetic ablation, conopeptides, which are natural and potent antagonists of N-type VGCCs have been used extensively in recent years (Olivera et al., 1987; Hopkins et al., 1995; Vanegas and Schaible, 2000; Lee, 2013). Relatedly, intrathecal administration of conopeptides is anti-nociceptive in acute inflammatory pain models (Olivera et al., 1987; Staats et al., 2004; Rigo et al., 2013). A recent study examined the efficacies of ω-conotoxin MVIIA and Phα1β in alleviating chemotherapy associated pain and reported positive outcomes (Rigo et al., 2013). Thus, in this study we likewise turned toward a pharmacological strategy with conotoxin to inhibit Cav2.2 activity during inflammatory states. We administered ω-conotoxin GVIA (300 pmol) in the lumbar space by intrathecal injection after CFA induced inflammation at regular intervals for 72 h. A significant reduction in thermal and cold pain sensitivities was observed, without effecting mechanical pain sensitivity. The sensory modality specific differences we observe by blocking Cav2.2 during inflammation, which differ from published reports, could be due to differences in the pharmacological properties of ω-conotoxin GVIA and ω-conotoxin MVIIA.

Sensory nerves involved in intra-plantar inflammation have cell bodies in the lumbar DRG. These DRGs process the signals received from their peripheral afferent projections and relay information to the CNS through the central projections in the dorsal horn of the spinal cord (Zhang and Dougherty, 2014). Persistent inflammation triggered by CFA injection or nerve injury generated via chronic constriction or nerve ligation, have been shown to amplify this signaling cascade (Lu and Gold, 2008; Lu et al., 2010). Intra-plantar injection of inflammatory soup in rats has been shown to increase the frequency of spontaneous neuronal discharge (Kasai and Mizumura, 2001; Ma et al., 2006). Multiple ion channels including Na^+^, K^+^ have been shown to contribute to the lowering of action potential threshold and resting membrane potential in lumbar DRGs, which affects a large population of afferent neurons. In agreement with these findings, our voltage-clamp and current clamp analyses carried out post CFA treatment with and without ω-conotoxin in lumbar (L4–L7) DRGs showed a significant increase in Cav2.2 current density and spontaneous action potentials, which were inhibited by ω-conotoxin treatment. These findings implicate a role for Cav2.2 in increased excitability of neurons after inflammation.

An emerging literature has shown that a variety of factors contribute toward increased primary afferent growth at the onset on inflammation including cytokines, fibronectin, and nerve growth factor (Woolf et al., 1997; Miller et al., 2009; Tonge et al., 2012). Studies in *Xenopus laevis* and the pheochromocytoma (PC12) cell line suggest that calcium influx through VGCC causes growth cone extension, increases in connectivity, and neurite outgrowth (Solem et al., 1995; Spitzer, 2006; Sann et al., 2008). This prompted us to analyze if there were any changes in nerve growth following CFA-induced inflammation. DRGs, sciatic nerve, and hind paws harvested 24 h post intra-plantar CFA injection revealed a significant increase in TUJ1, a neuronal marker which labels neurites. In addition, a robust increase in Cav2.2 surface expression in DRGs was also seen post inflammation. This led us to hypothesize that Cav2.2 might be driving neurite outgrowth. Remarkably, a significant reduction in the neuritogenesis marker TUJ1 was observed post ω-conotoxin GVIA treatment across DRGs, sciatic nerve, and paws. This suggests a direct correlation between Cav2.2 activity and neuronal growth in peripheral inflammation.

Peripheral inflammation has been shown to activate immediate early genes like cFos (Xing et al., 1996; Sgambato et al., 1998) acutely and the ERK1/2-MAPK on a longer timescale (Ji et al., 2002). The functional role of ERK activation in neuronal physiology is very broad and includes neurite outgrowth. Elevated intracellular Ca^2+^ triggers various signaling pathways including protein kinases such as Calmodulin-dependent kinases (CaMK) and ERKs in neuroblastoma cell line. Additionally, ERK kinase inhibitor blocks BDNF-induced neurite outgrowth in neuroblastoma cell line SH-SY5Y (Encinas et al., 1999). Here, we showed a significant reduction in phosphorylated ERK (pERK1/2) upon ω-conotoxin treatment in primary afferents. Therefore, in addition to inhibiting neuritogenesis during inflammation, blockade of Cav2.2 might be downregulating the ERK1/2-MAPK signaling cascade and alleviating inflammatory pain hypersensitivity. In addition, a recent HA-tagged Cav2.2 knock-mouse was generated and it was shown that Cav2.2 is expressed in the dorsal horn of the spinal cord in close apposition to terminal endings of nociceptive neurons (Nieto-Rostro et al., 2018). Therefore, it is possible that in addition the peripheral changes we have demonstrated with Cav2.2 in this work, spinal cord changes in Cav2.2 may also drive components of inflammation induced hyperalgesia. Taken together, this work adds a new paradigm to what changes may be driving inflammation induced thermal hyperalgesia and suggests that strategies aimed at inhibiting neurite growth may serve as potent analgesics to block some forms of pain.

## Data Availability

All datasets generated for this study are included in the manuscript and/or the Supplementary Files.

## Ethics Statement

The animal study was reviewed and approved by North Carolina State University and the University of Pennsylvania Institutional Animal Care and Use Committee.

## Author Contributions

SP and LM designed and carried out the experiments. IA-S and SM designed the experiments. All authors contributed to the writing of the manuscript.

## Conflict of Interest Statement

The authors declare that the research was conducted in the absence of any commercial or financial relationships that could be construed as a potential conflict of interest.

## Supplementary Material

FIGURE S1 Co-expression of Cav2.2 and the gentle touch sensor MrgprB4: Overlap of Cav2.2 and MrgprB4 with and without CFA injury. Contralateral DRGs **(A–C)** and Ipsilateral DRGs **(D–F)** show no significant change Cav2.2-MrgprB4 mRNA co-expression.
