# Supplementary figures and images for: Inflammation Induced Sensory Nerve Growth and Pain Hypersensitivity Requires the N-Type Calcium Channel Cav2.2
Source: Front Neurosci. 2019 Sep 19;13:1009. doi: 10.3389/fnins.2019.01009 (PMC6761232; doi:10.3389/fnins.2019.01009)

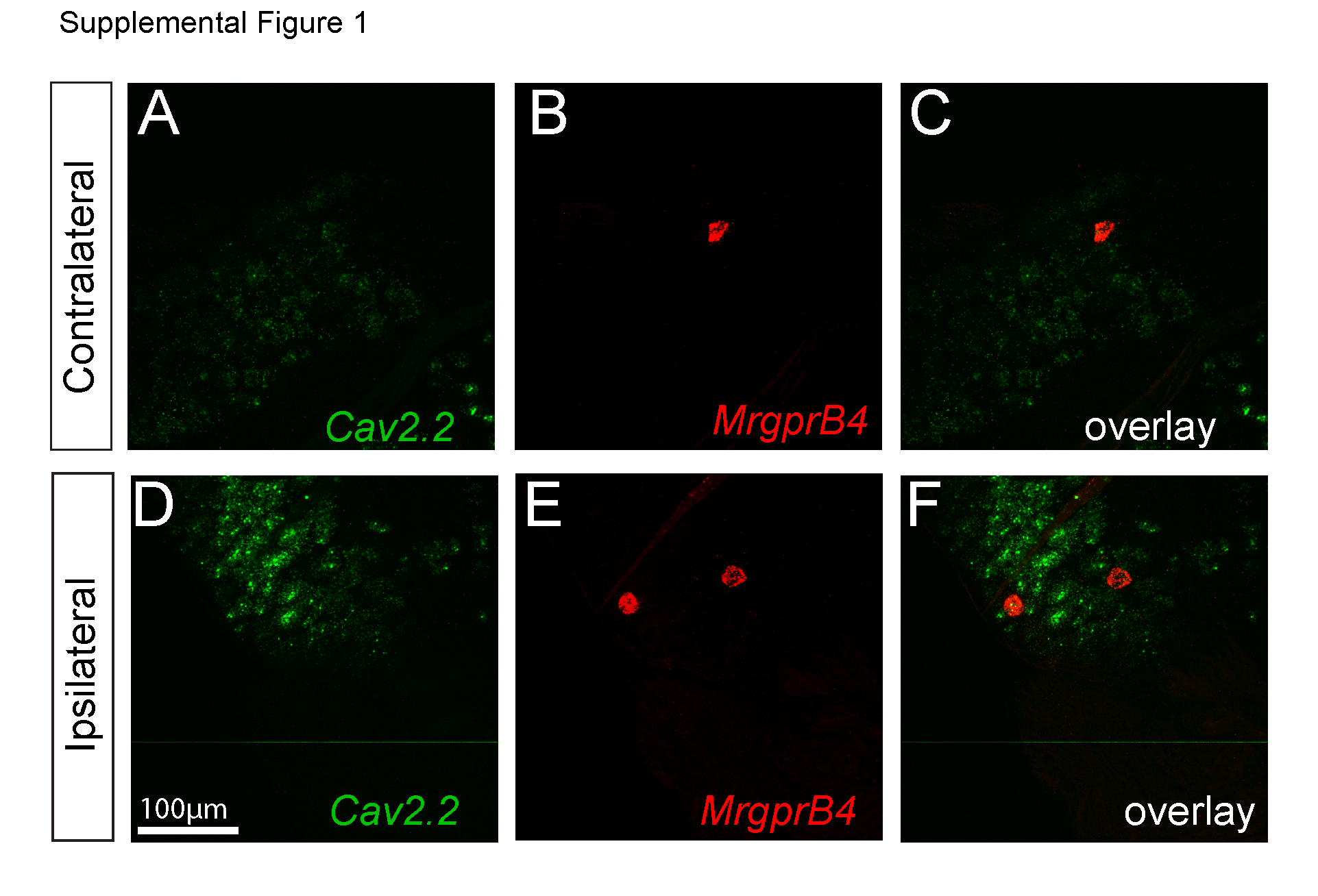

Supplement: FIGURE S1 — Co-expression of Cav2.2 and the gentle touch sensor MrgprB4: Overlap of Cav2.2 and MrgprB4 with and without CFA injury. Contralateral DRGs (A–C) and Ipsilateral DRGs (D–F) show no significant change Cav2.2-MrgprB4 mRNA co-expression. [file Image_1.TIFF]
